# Supplementary material for: Exploiting mechanisms for hierarchical branching structure of lung airway
Source: PLoS One. 2024 Aug 30;19(8):e0309464. doi: 10.1371/journal.pone.0309464 (PMC11364422; doi:10.1371/journal.pone.0309464)
Supplement: S1 Fig — ROIs are indicated by yellow circles on the bright-field images. 2D tissue curvature and ERK activity were obtained from a series of ROI clockwise along the epithelial cross-section. (PDF) [file pone.0309464.s001.pdf]

S1 FIG

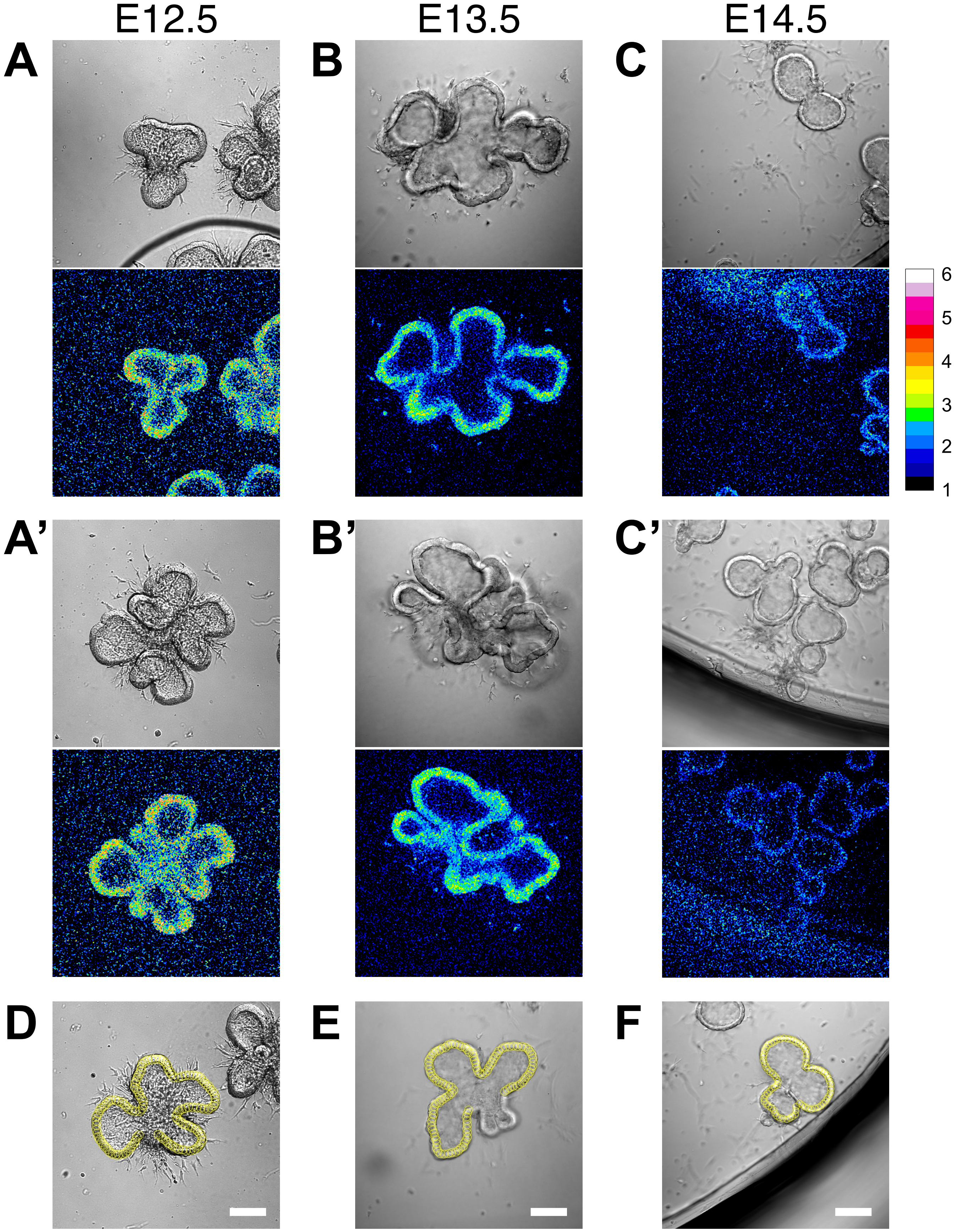

S1 Fig. Bright-field images of embryonic lung epithelium and the corresponding ERK activity. (A–C, A'–C) Examples of ERK activity map of epithelial explants. A' and A, B and B', C and C' show different samples observed under the same conditions as A, B and C in Fig 1, respectively. These results indicate that the strength of the signal response in the epithelium and the dispersion of the signal in the tissue weaken with development. **(D–E)** ROIs used to obtain the ERK activity map in Fig 1. ROIs are indicated by yellow circles on the bright-field images. 2D tissue curvature and ERK activity were obtained from a series of ROI clockwise along the epithelial cross-section.
