# Supplementary material for: Exploiting mechanisms for hierarchical branching structure of lung airway
Source: PLoS One. 2024 Aug 30;19(8):e0309464. doi: 10.1371/journal.pone.0309464 (PMC11364422; doi:10.1371/journal.pone.0309464)
Supplement: S2 Fig — (A) The heat maps of the ERK activity (upper panels) and bright field images (lower panels) for E13.5 and E14.5 cysts. The ERK activity quantified for the cyst is indicated on each panel. The epithelial thickness seems different between in E13.5 and E14.5, and the statistical analysis was demonstrated in S3 Fig. (B) The fluorescent signals of FGF10-Alexa488 (upper panels) and bright field images (lower panels) for E13.5 and E14.5 explants. High backgrounds of the fluorescent images were caused by the high FGF10-Alexa488 concentration in the Matrigel and the medium. The mean fluorescent intensity is indicated on each panel. Scale bar: 50 μm. (PDF) [file pone.0309464.s002.pdf]

S2 FIG

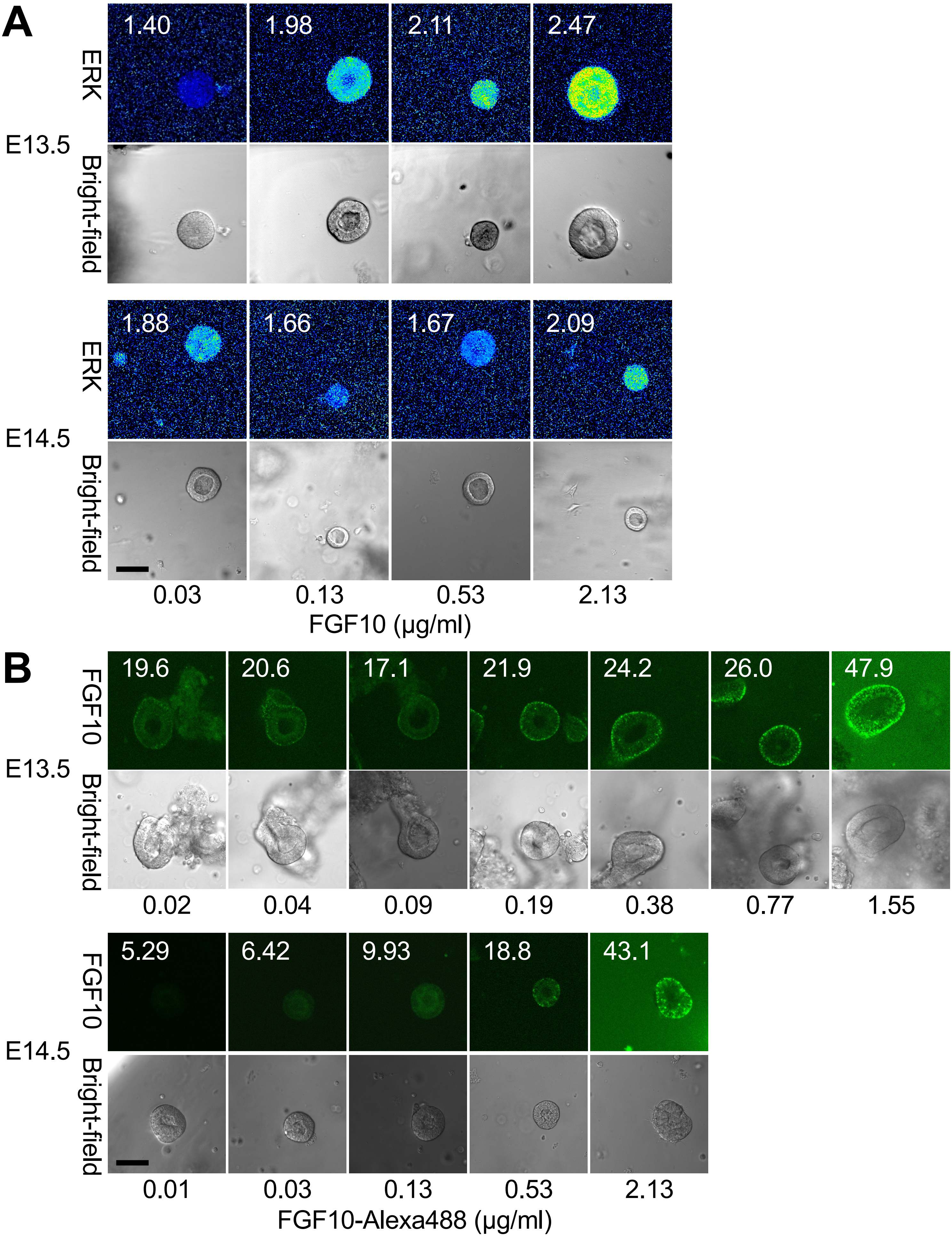

A
E13.5
ERK
Bright-field
1.40
1.98
2.11
2.47
E14.5
ERK
Bright-field
1.88
1.66
1.67
2.09
0.03
0.13
0.53
2.13
FGF10 (µg/ml)
B
E13.5
FGF10
Bright-field
19.6
20.6
17.1
21.9
24.2
26.0
47.9
0.02
0.04
0.09
0.19
0.38
0.77
1.55
E14.5
FGF10
Bright-field
5.29
6.42
9.93
18.8
43.1
0.01
0.03
0.13
0.53
2.13
FGF10-Alexa488 (µg/ml)

S2 Fig. Representative images in the ERK activity measurement in E13.5 and E14.5 lung epithelial explants in Fig 2. (A) The heat maps of the ERK activity (upper panels) and bright field images (lower panels) for E13.5 and E14.5 cysts. The ERK activity quantified for the cyst is indicated on each panel. The epithelial thickness seems different between in E13.5 and E14.5, and the statistical analysis was demonstrated in Fig S3. **(B)** The fluorescent signals of FGF10-Alexa488 (upper panels) and bright field images (lower panels) for E13.5 and E14.5 explants. High backgrounds of the fluorescent images were caused by the high FGF10-Alexa488 concentration in the Matrigel and the medium. The mean fluorescent intensity is indicated on each panel. Scale bar: 50 μm.
