# Supplementary material for: Exploiting mechanisms for hierarchical branching structure of lung airway
Source: PLoS One. 2024 Aug 30;19(8):e0309464. doi: 10.1371/journal.pone.0309464 (PMC11364422; doi:10.1371/journal.pone.0309464)
Supplement: S3 Fig — ROIs for thickness measurement. Small circles were fitted to the local thickness of the epithelium. The mean and standard deviation of the circle diameters were calculated to represent the thickness. (PDF) [file pone.0309464.s003.pdf]

S3 FIG

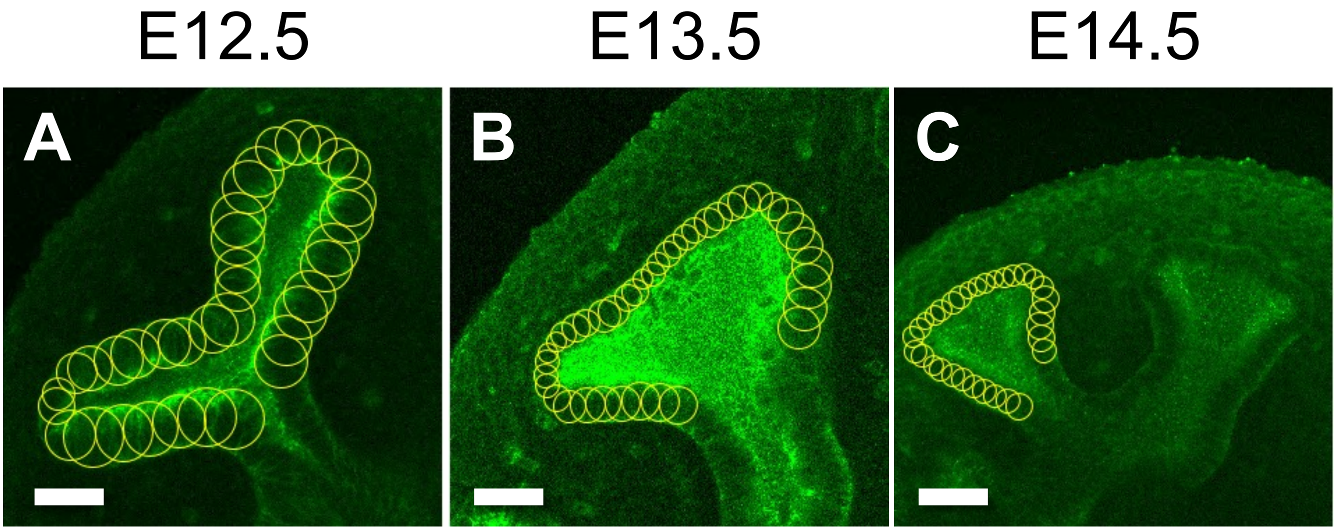

S3 Fig. ROIs used to measure the epithelial thickness in Fig 6. ROIs for thickness measurement. Small circles were fitted to the local thickness of the epithelium. The mean and standard deviation of the circle diameters were calculated to represent the thickness.
