# Supplementary material for: Exploiting mechanisms for hierarchical branching structure of lung airway
Source: PLoS One. 2024 Aug 30;19(8):e0309464. doi: 10.1371/journal.pone.0309464 (PMC11364422; doi:10.1371/journal.pone.0309464)
Supplement: S4 Fig — The E13.5 epithelial cyst was cultured with the FGF10-Alexa488 and subsequently applied to the immunostaining of FGF10, FGFR2, and heparan sulfate. The samples were fixed with 2% paraformaldehyde in PBS for 5 min on ice. We used anti-FGF10 antibody (H-121, 1:20; Santa Cruz), anti-Bek (C-17, 1:100; Santa Cruz) for FGFR2 detection, and anti-heparan sulfate (10E4, 1:100; Seikagaku). The second antibodies were Alexa Fluor 568- conjugated goat anti-rabbit IgG and goat anti-mouse IgG (1:200; Thermo Fisher). The Alexa488 signals in cells was diffused but remained after the staining. The result of FGF10 immunostaining coincided with the FGF10-Alexa488 signal, except for that Matrigel containing FGF10-Alexa488 strongly reacted to anti-FGF10 antibody (upper panels). It was also found that FGFR2 mostly colocalized to the FGF10-Alexa488 signals (middle panels). Heparan sulfate is known to be localized to the cell surface and to support the FGF10-FGFR2 binding (lower panels). Scale bar: 50 μm. (PDF) [file pone.0309464.s004.pdf]

S4 FIG

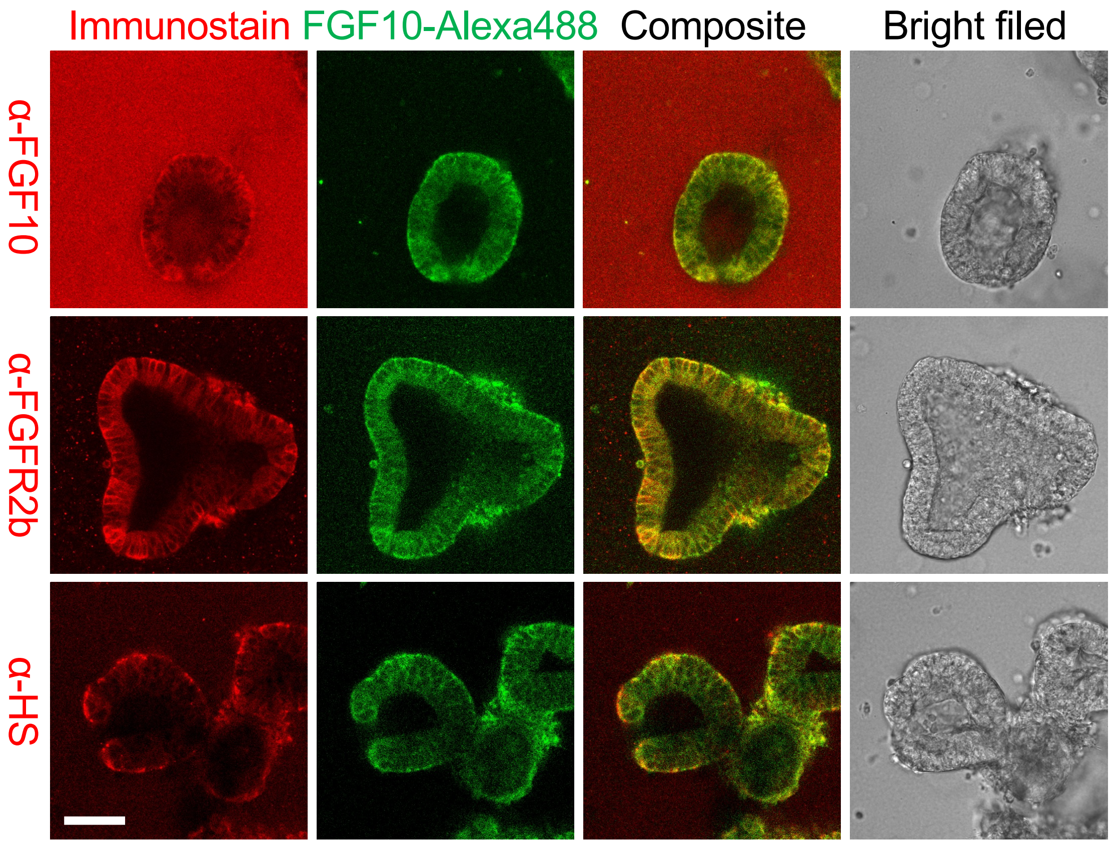

S4 Fig. Immunohistological analysis validating the FGF10-Alexa488 uptake experiment as the indication of the FGF10-FGFR2 internalization. The E13.5 epithelial cyst was cultured with the FGF10-Alexa488 and subsequently applied to the immunostaining of FGF10, FGFR2, and heparan sulfate. The samples were fixed with 2% paraformaldehyde in PBS for 5 min on ice. We used anti-FGF10 antibody (H-121, 1:20; Santa Cruz), anti-Bek (C-17, 1:100; Santa Cruz) for FGFR2 detection, and anti-heparan sulfate (10E4, 1:100; Seikagaku). The second antibodies were Alexa Fluor 568-conjugated goat anti-rabbit IgG and goat anti-mouse IgG (1:200; Thermo Fisher). The Alexa488 signals in cells was diffused but remained after the staining. The result of FGF10 immunostaining coincided with the FGF10-Alexa488 signal, except for that Matrigel containing FGF10-Alexa488 strongly reacted to anti-FGF10 antibody (upper panels). It was also found that FGFR2 mostly colocalized to the FGF10-Alexa488 signals (middle panels). Heparan sulfate is known to be localized to the cell surface and to support the FGF10-FGFR2 binding (lower panels). Scale bar: 50 μm.
