# Supplementary material for: Exploiting mechanisms for hierarchical branching structure of lung airway
Source: PLoS One. 2024 Aug 30;19(8):e0309464. doi: 10.1371/journal.pone.0309464 (PMC11364422; doi:10.1371/journal.pone.0309464)
Supplement: S5 Fig — The cysts from E13.5 (A) and E14.5 (B, C, and D) mice were cultured in the FGF10-supplimented Matrigel for 18 h (A and C) or 3 h (B and D), following the procedures outlined in in Fig 2 and S2 Fig. The series for E13.5 and E14.5 are presented to the right of (A) and (D), respectively. For E13.5, morphometric analysis could not be conducted on samples cultured for 3 h with FGF and for 18 h without FGF, as cyst formation in these cases showed minimal progress. Cyst thickness was quantified by estimating outer and inner diameters in cross-sections using Fiji (NIH), calculated as the difference between them. The statistical analysis was performed by using R. The significant difference among the linear regression lines was tested by the analysis of covariance (ANOVA). (A) E13.5 cysts cultured for 18 h. The thickness-to- diameter correlations in four different doses of FGF10 showed no significant difference. The linear regression for the pooled data is y = 0.10x + 11.39 with R2 = 0.24 (black line), suggesting that epithelia tend to be thicker in larger cysts. Small explants may have been obtained by tearing off the tip of the epithelium where the epithelium is thin, while large explants may have been from a large area containing a thicker duct. (B) E14.5 cysts cultured for 3 h. The correlations in five different doses showed no significant difference. The linear regression for the pooled data is y = 0.16x + 9.48 with R2 = 0.48 (red dashed line), confirming the same tendency in (A). (C) E14.5 cysts cultured for 18 h. The correlation in five different doses showed no significant difference except for the samples without FGF10 (P < 0.001), suggesting that FGF10 exposure flattened the epithelium. The linear regression for the pooled data of the FGF10 exposed cases is y = 0.10x + 9.48 with R2 = 0.48 (blue line), and for the case without FGF10 y = 0.21x + 8.24 with R2 = 0.79 (blue dotted line). Both correlations were significantly different from the case of 3-h culture in (B) ( [file pone.0309464.s005.pdf]

S5 FIG

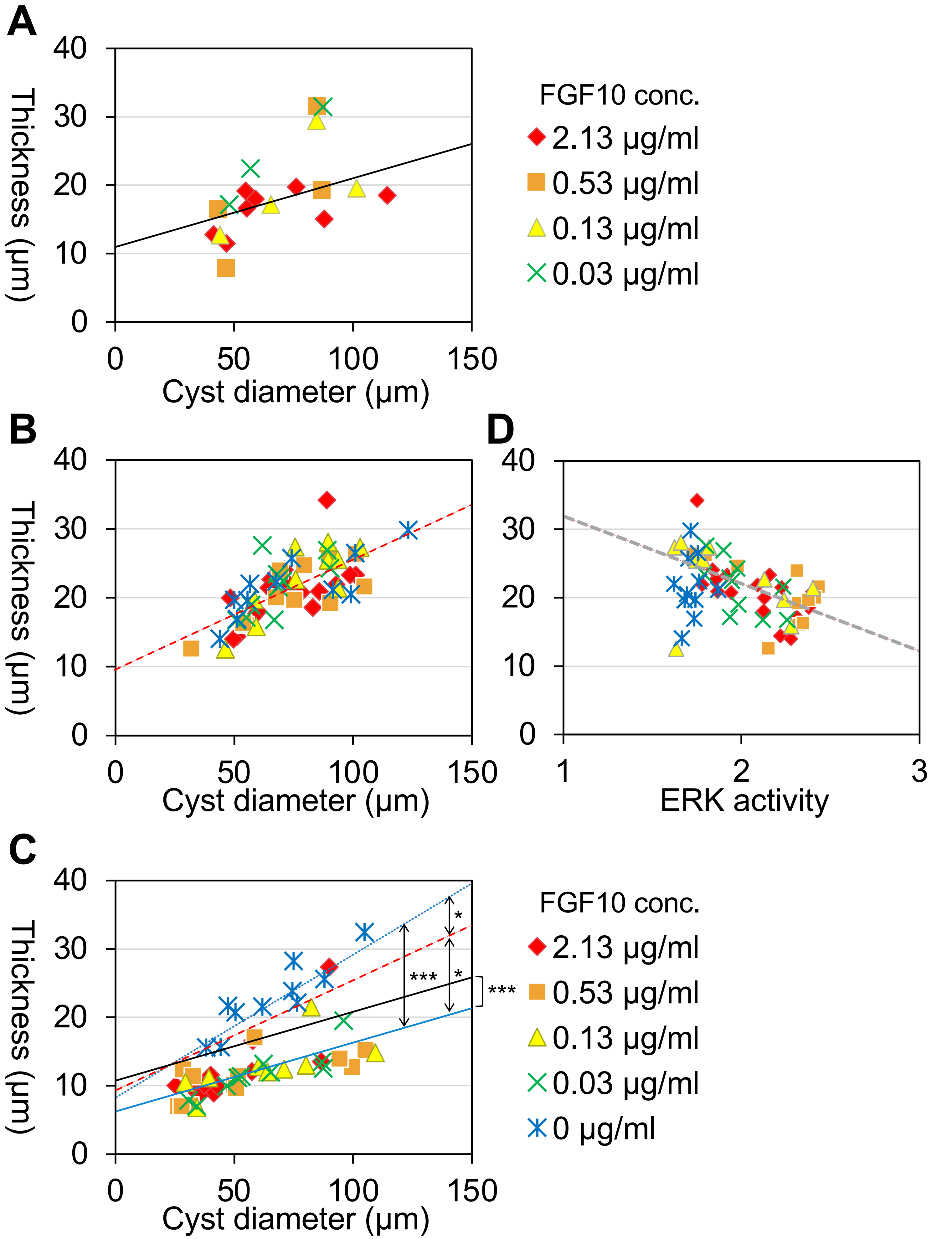
A
B
C
D
Thickness (µm)
Cyst diameter (µm)
ERK activity
0
10
20
30
40
50
100
150
1
2
3
FGF10 conc.
2.13 µg/ml
0.53 µg/ml
0.13 µg/ml
0.03 µg/ml
0 µg/ml
*
***

S5 Fig. Shape changes and ERK activity of lung epithelial cysts. The cysts from E13.5 (A) and E14.5 (B, C, and D) mice were cultured in the FGF10-supplimented Matrigel for 18 h (A and C) or 3 h (B and D), following the procedures outlined in in Fig 2 and S2. The series for E13.5 and E14.5 are presented to the right of (A) and (D), respectively. For E13.5, morphometric analysis could not be conducted on samples cultured for 3 h with FGF and for 18 h without FGF, as cyst formation in these cases showed minimal progress. Cyst thickness was quantified by estimating outer and inner diameters in cross-sections using Fiji (NIH), calculated as the difference between them. The statistical analysis was performed by using R. The significant difference among the linear regression lines was tested by the analysis of covariance (ANOVA). **(A)** E13.5 cysts cultured for 18 h. The thickness-to-diameter correlations in four different doses of FGF10 showed no significant difference. The linear regression for the pooled data is $y = 0.10x + 11.39$ with $R^2 = 0.24$ (black line), suggesting that epithelia tend to be thicker in larger cysts. Small explants may have been obtained by tearing off the tip of the epithelium where the epithelium is thin, while large explants may have been from a large area containing a thicker duct. **(B)** E14.5 cysts cultured for 3 h. The correlations in five different doses showed no significant difference. The linear regression for the pooled data is $y = 0.16x + 9.48$ with $R^2 = 0.48$ (red dashed line), confirming the same tendency in (A). **(C)** E14.5 cysts cultured for 18 h. The correlation in five different doses showed no significant difference except for the samples without FGF10 ($P < 0.001$), suggesting that FGF10 exposure flattened the epithelium. The linear regression for the pooled data of the FGF10 exposed cases is $y = 0.10x + 9.48$ with $R^2 = 0.48$ (blue line), and for the case without FGF10 $y = 0.21x + 8.24$ with $R^2 = 0.79$ (blue dotted line). Both correlations were significantly different from the case of 3-h culture in (B) ($P < 0.05$), confirming that FGF10 exposure progressively flattened the epithelium, whereas prolonged culture without FGF10 thickened the epithelium. These correlations are also significantly different from the case of E13.5 cysts in (A), supporting the difference of the epithelial properties along the developmental process. The slopes in (A) and pooled data in (B) did not differ, whereas their intercepts significantly differ by 5.14 ($P < 0.001$), which agrees with that epithelium in the earlier stage is thicker. **(D)** Thickness was plotted against ERK activity for the corresponding sample in (B). The linear regression for the pooled data of the FGF10 exposed cases is $y = -10.08x + 42.10$ with $R^2 = 0.28$ (gray dotted line), indicating that thinner epithelia exhibit higher activity. No significant correlation was found for the samples without FGF10.
