# Supplementary material for: Exploiting mechanisms for hierarchical branching structure of lung airway
Source: PLoS One. 2024 Aug 30;19(8):e0309464. doi: 10.1371/journal.pone.0309464 (PMC11364422; doi:10.1371/journal.pone.0309464)
Supplement: S1 Table — (PDF) [file pone.0309464.s011.pdf]

S1 Table

Parameter and description		Fig 3C	Fig 3D
k_{p}	Angle dependence of cell division	0.008	*
θ_{p}	Minimum angle for cell division	0.2	0.2
$\hat{p}$	Basic probability	0.001	0.001
k_{m}	Angle dependence of active migration	25	*
k_{wall}	Interaction between adjacent cells [42]	60	60
k_{bend}	Bending rigidity along the cell sequence [42]	15	15
γ	Friction coefficient for cell displacement	15	15

*: Values are indicated in the figure.
