# Supplementary material for: Exploiting mechanisms for hierarchical branching structure of lung airway
Source: PLoS One. 2024 Aug 30;19(8):e0309464. doi: 10.1371/journal.pone.0309464 (PMC11364422; doi:10.1371/journal.pone.0309464)
Supplement: S2 Table — (PDF) [file pone.0309464.s012.pdf]

S2 Table

Parameter and description		Fig 4C	Fig 4D	Fig 5A
k_{q}	Dependency of cell division on the angle of the basal contour	0.9	0.9	0
φ_{q}	Minimum angle for cell division	0.5	0.5	0
$\hat{q}$	Basic probability for cell division	0.0001	0.0001	0
k_{w}	Dependency of active migration on the angle of the basal contour	1	1	0
φ_{w}	Minimum angle for the active migration	0.7	0.7	0
$\bar{a}$	Optimal apical length of the cell [36]	0	0	0
$\bar{b}$	Optimal basal length of the cell [36]	1	1	1
$\bar{s}$	Optimal area of the cell	1	1	1
k_{a}	Apical length regulatory coefficient [36]	0	1	**
k_{b}	Basal length regulatory coefficient [36]	300	300	300
k_{s}	Area regulatory coefficient	3	3	3
k_c	Lateral length regulatory coefficient [36]	0	0	0
k_{ab}	Cell shape symmetry coefficient [36]	10	10	5
k_{bend}	Bending rigidity of the basal contour [36]	0.2	0.2	2
γ	Friction coefficient	20	20	20

**: The value is 0 during relaxation without apical constriction and 3 for applying apical constriction.
