# Supplementary material for: Exploiting mechanisms for hierarchical branching structure of lung airway
Source: PLoS One. 2024 Aug 30;19(8):e0309464. doi: 10.1371/journal.pone.0309464 (PMC11364422; doi:10.1371/journal.pone.0309464)
Supplement: S3 Table — (PDF) [file pone.0309464.s013.pdf]

S3 Table

Parameter and description		Fig 7C	Fig 7D	Fig 7E	Fig 7F	Fig 7G
φ_{d}	Critical angle for cell type switching	-0.45	-0.3	-0.3	-0.45	-0.3
k_{μ}	Active migration coefficient for tip type	0.8	0.6	0.6	0.8	0.6
ρ	Cell division probability for tip type	0.00025	0.0002	0.0002	0.00025	0.0002
$\bar{a}_{\mathrm{t}}$	Optimal apical length [36] for tip type	0	0	0	0	0
$\bar{a}_{\mathrm{d}}$	Optimal apical length [36] for duct type	1.1	1.4	1.1	1.1	1.4
$\bar{b}$	Optimal basal length [36]	1.1	1.4	1.1	1.1	1.4
$\bar{s}_{\mathrm{t}}$	Optimal cell area for tip type	1	1	1	1	1
$\bar{s}_{\mathrm{d}}$	Optimal cell area for duct type	2.7	2.7	2.7	2.7	2.7
k_{at}	Apical length regulatory coefficient [36] for tip type	70	70	70	35	35
k_{ad}	Apical length regulatory coefficient [36] for duct	40	40	40	40	40
k_{b}	Basal length regulatory coefficient [36]	300	300	300	300	300
k_{s}	Area regulatory coefficient	50	50	50	50	50
k_{c}	Lateral length regulatory coefficient [36]	0	0	0	0	0
k_{ab}	Cell shape symmetry coefficient [36]	200	200	200	200	200
k_{bend}	Bending rigidity of the basal side	30	30	30	30	30
γ	Friction coefficient	20	20	20	20	20
